# Exogenous Fungal Endophthalmitis: Clues to *Aspergillus* Aetiology with a Pharmacological Perspective

**DOI:** 10.3390/microorganisms9010074

**Published:** 2020-12-30

**Authors:** Tommaso Lupia, Silvia Corcione, Antonio Maria Fea, Michele Reibaldi, Matteo Fallico, Francesco Petrillo, Marilena Galdiero, Silvia Scabini, Maria Sole Polito, Umberto Ciabatti, Francesco Giuseppe De Rosa

**Affiliations:** 1 Department of Medical Sciences, University of Turin, 10124 Turin, Italy; tommaso.lupia89@gmail.com (T.L.); corcione.silvia@gmail.com (S.C.); silvia.scabini@unito.it (S.S.); francescogiuseppe.derosa@unito.it (F.G.D.R.); 2 Department of Surgical Sciences, Eye Clinic Section, University of Turin, 10124 Turin, Italy; antoniomfea@gmail.com (A.M.F.); mreibaldi@libero.it (M.R.); msolepolito@gmail.com (M.S.P.); umberto.ciabatti@hotmail.it (U.C.); 3 Department of Ophthalmology, University of Catania, 95123 Catania, Italy; matteofallico@hotmail.com (M.F.); francescopetrillo09@gmail.com (F.P.); 4 Department of Experimental Medicine, University of Campania “Luigi Vanvitelli”, 80138 Naples, Italy

**Keywords:** endophtalmitis, fungal endophtalmitis, aspergillus, antifungal therapy

## Abstract

Exogenous fungal endophthalmitis (EXFE) represents a rare complication after penetrating ocular trauma of previously unresolved keratitis or iatrogenic infections, following intraocular surgery such as cataract surgery. The usual latency period between intraocular inoculation and presentation of symptoms from fungal endophthalmitis is several weeks to months as delayed-onset endophthalmitis. *Aspergillus* spp., is the most common causative mould pathogen implicated in this ocular infection and early diagnosis and prompt antimicrobial treatment, concomitantly in most cases with expert surgical attention, reduce unfavorable complications and increase the possibility of eye function preservation. Topical, intravitreal and systemic antifungal molecules are the mainstay of a medical approach to the disease and azoles, polyenes and in particular cases echinocandins are the pharmacological classes most commonly used in clinical practice. This review discusses pharmacokinetics and pharmacodynamic of antifungal agents in their principal modes of administration with a focus on their ability to achieve high drug concentration in the vitreous and ocular tissues.

## 1. Introduction

The term “endophthalmitis” is referred to one of the most striking eye infections due to infection of the ocular cavity and adjacent structures by fungi and bacteria. Most cases of endophthalmitis are exogenous, in which pathogens from an external source or on the ocular surface, are introduced into the eye. Exogenous endophthalmitis (EE) account for 85% to 98% of all cases of endophthalmitis [1]. Despite the burden of fungal aetiology in this field being small, this type of infection is often associated with poor visual outcomes, being influenced by climate conditions and mode of infection [1,2,3,4]. According to *Rychener* classification [5], exogenous fungal endophthalmitis (EXFE) occurs as a result of extension of keratomycosis, eye surgery, or penetrating ocular trauma. Fungal endophthalmitis accounts increased, over the last 20 years, from 8.6% to 18.6% of culture-positive cases. The clinical presentation of *Aspergillus*-EXFE may vary from an indolent, mild external disease to fulminant, necrotizing destruction of the globe [1]. Asian studies have reported fungi as the causative organisms in approximately 11.1% to 17.54% of total cases of EE, with the rest being attributed to bacterial causes [6]. *Aspergillus* is a saprophyte fungus, and it is present everywhere [7]. *Aspergillus* commonly infects the lungs and the paranasal sinuses. Furthermore, it can rarely cause tear duct infections and prolonged local therapy with antibiotics and corticosteroids is a high risk factor [8]. Therefore, in early onset endophthalmitis, *Aspergillus* infection should be considered in differential diagnosis with bacterial endophthalmitis, especially in tropical climates [1,2]. In most cases, the dominant *Aspergillus* subspecies responsible for EFE were *A. fumigatus*, *A. flavus*, *A. niger*, *A. nidulans and A. terreus*. Other species detected in ophthalmic disease are *A. glaucus*, *A. ustus*, *A. terreus* and *A. versicolor* [3,4]. Wykoff et al. (2008) reported the differences between the clinical categories of exogenous fungal endophthalmitis. Culture-positive exogenous fungal endophthalmitis occurred in 41 eyes, including 35 cases (85%) associated with filamentous fungi and 6 cases (15%) regarded *Candida* species Although *Fusarium* was correlated with most keratitis cases (13 of 18; 72%), while Aspergillus was detected in postoperative cases (5 of 13; 38%), 18 cases (44%) associated with fungal keratitis, 10 cases (24%) were correlated with penetrating ocular trauma, and 13 cases (32%) with intraocular surgery [9]. A recent study including 91 patients with culture-proven *Aspergillus* endophthalmitis showed that trauma was the most common cause of EXFE and that *A. flavus* (34, 1%) was the predominant infecting species [10]. Early diagnosis and aggressive treatment are the key for better visual outcomes, but proven diagnosis is troublesome and therapeutic options are scarce [1,2,3,4,5]. We aimed to review current knowledge about EXFE due to *Aspergillus* spp. together with a focus on the pharmacokinetics (PK) and pharmacodynamics (PD) of antifungal agents in their principal route of administration.

## 2. Materials and Methods

**Figure 1:**
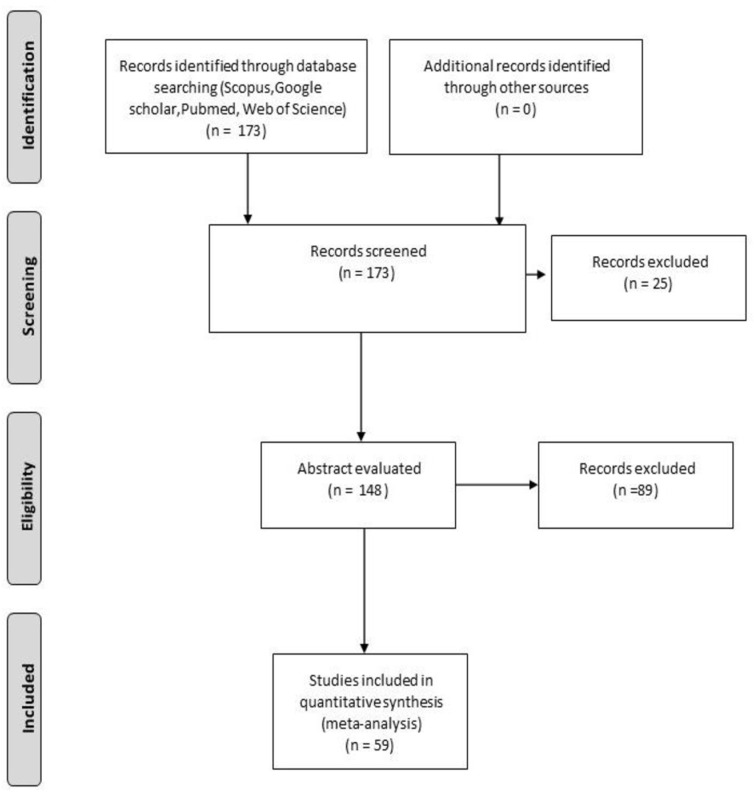
PRISMA flow diagram, showing the process of study selection.

This study provides an state-of-the-art review of the published literature encompassing the involvement of Aspergillus in EXFE. Different electronic resources were employed to perform the literature search, including Scopus, Google scholar, PubMed and Web of Science. The keywords used have been: “exogenous endophthalmitis”, “Aspergillus and endophthalmitis”, while the criteria for selecting articles were “studies reported in English because of language barriers”. Two investigators (TL and SS) reviewed the identified articles, initially by title and abstract and then in detail, using a customized data abstraction form. Studies with wrong subject matter and duplications were excluded from our analysis. The results returned 173 published papers up to the 2020. Of these articles, 59 were selected, summarized, and critically discussed so as to provide a consistent review. Figure 1 illustrates the PRISMA flow diagram for study selection. Given the nature of the review, no ethics approval was required.

## 3. Epidemiology and Risk Factors

### 3.1. Endophthalmitis after Ocular Surgery or Invasive Procedures

#### 3.1.1. Endophthalmitis Post-Cataract Surgery

The incidence of post-cataract endophthalmitis is rare, ranging from 0.03% to 0.2% and the majority of them are caused by bacteria [7,8,9,10,11,12,13,14]. Among fungi, *Aspergillus* spp. is the most common reported after cataract surgery, followed by *Fusarium* spp. with recent reports of isolated outbreaks [11,12,13,14,15,16,17,18]. During the last years, new approaches in cataract surgery, from intracapsular cataract extraction to laser-assisted surgery, led to less invasive surgical methods (e.g., microincisions, injectable lenses, topical anaesthesia and sutureless surgical wounds), thus reducing the rate of post-operative endophthalmitis [1,2,3,4]. On the other hand, in place of silicone intraocular lenses, the absence of intracameral antibiotic administration, occurrence of intraoperative complications and old age can increase the risk of ocular infections [3,4,5,6,7,8,9]. Fungal endophthalmitis after cataract surgery is more prevalent in developing countries such as China and India, where up to 12.7% and 21.8% of the cases, respectively, were attributed to fungi [9,14,18]. In the US the rate of fungal endophthalmitis after cataract surgery is low, ranging from 0.002% to 0.005% [12,18]. In a study conducted by Sen et [19], 17 patients with culture-proven fungal endophthalmitis after cataract surgery were evaluated including intravitreal antibiotics and antifungals, pars plana vitrectomy (PPV), intraocular lens explantation (IOL) and scleral fixated IOL implantation (SFIOL). Following the assessment of visual acuity the presence of *A. terreus* and corneal involvement in addition to endophthalmitis have been found to be prognostic markers [19].

#### 3.1.2. Endophthalmitis Post-Vitrectomy

Several pieces of evidence in literature support the use of pars plana vitrectomy to manage fungal endophthalmitis. Vitrectomy can increase the likelihood of establishing a proper diagnosis, of improving treatment of infection by removing fungal elements in the vitreous. Moreover, vitrectomy can be a useful aid in the removal of other structures intraocularly inoculated and is an important tool in the management of infectious complications that can lead to detachment of the retina and epiretinal membrane [20]. Mould infections after vitrectomy remain a rare event with high variability between temperate to tropical zones, and high heterogeneity of epidemiological data between hospitals [21]. In a single, tertiary eye care in India [22], of 111,876 pars plana vitrectomy (PPV) performed, 45 cases developed acute onset postoperative endophthalmitis. Among the microorganisms isolated in the 24 culture-positive cases, *Aspergillus* was the only fungus isolated (5/24; 20.8%). Conversely, the article compared the incidence rates of endophthalmitis in both 20 G PPV and mini-invasive approach PPV, demonstrating a higher incidence of endophthalmitis in 20 G PPV (0.057% vs. 0.012%) [22]. The same study suggests a protective role of intraocular tamponade [21]. Dave et al. [23] collected data from four tertiary eye cares in India, with 38,591 patients undergoing PPV between 1990 and 2014: the clinical incidence of post-vitrectomy endophthalmitis was 0.052%, and culture-positive incidence was 0.031% with no *Aspergillus* spp. cases [23]. Similarly, in a 20-year study (1984–2003) in US [24] the incidence of endophthalmitis after PPV was about 0.039% and no *Aspergillus* spp. infections occurred.

#### 3.1.3. Endophthalmitis Post-Intravitreal Injection

The incidence of endophthalmitis as a consequence of intravitreal injection has been recognized to be in the range from 0.016% to 0.053%, according to several published studies [25]. The rates are higher after intravitreal corticosteroids than after intravitreal anti-VEGF agents [26]. While prophylaxis with topical antibiotics has been shown to increase, rather than reduce, the risk of post-injection endophthalmitis [27], preoperative disinfection with topical 5% povidone iodine represents the most commonly used and safest method against endophthalmitis [28,29]. Incidence of endophthalmitis does not seem to be affected by the type of intravitreal anti-VEGF drugs [25,26]. No case of Aspergillus spp. after anti-VEGF or corticosteroids intravitreal administration was reported at the time of writing; nevertheless, the rate of culture-negative suspected infections remains high.

#### 3.1.4. Endophthalmitis Post-Keratoplasty

Fungal infection following both lamellar and penetrating keratoplasty are most commonly caused by *Candida* spp. and only rarely by *Aspergillus* spp. [30,31]. As a matter of fact, a recent retrospective cohort study including 3069 patients who underwent penetrating and lamellar keratoplasty reported only 3 cases of EXFE, none of which caused by Aspergillus spp. [30]. A study conducted by Alharbi et al. [32] to identify the causative organisms of post-keratoplastic endophthalmitis evidenced that the review of charts of all patients with endophthalmitis diagnosis after keratoplasty in a tertiary hospital between January 1990 and January 2007, endophthalmitis developed in 55 cases in the penetrating keratoplasty group and the majority of isolated microbes were Gram positive bacteria (86.3%) [32]. Microbiology, as above mentioned, tends to vary worldwide [30,31]. Of 124 cases of fungal endophthalmitis post-keratoplasty reported in Saudi Arabia, the most common isolated organisms were Aspergillus spp. (29.8%) [31]. Isolated clinical cases on infection supported by *A. flavus* and *A. niger* were also reported in Italy and in Asian and Middle East countries [33,34].

#### 3.1.5. Epidemiology of Endophthalmitis after Keratomycosis

Fungal keratitis is a widely distributed infection of the cornea caused by a broad-spectrum of filamentous fungi and yeasts with annually increasing incidence. Incidence of endophthalmitis after keratomycosis was estimated to range from 0.5% to 6.3%, with an evisceration rate of 31% to 62.2% [35,36]. The main causative microorganisms, among moulds, were Fusarium and Aspergillus [2,3,4]. Shen et al. analyzed 10 cases of post keratitis endophthalmitisand isolated Aspergillus spp. in two out of ten cases [37]. Similarly, Wykoff et al., evaluated the microbiological pattern of 41 eyes affected by culture-positive fungal endophthalmitis [1]. Eighteen out of 41 EXFE were complications of a fungal keratitis [1]. Among them, only 6% were caused by *Aspergillus* spp. [9].

#### 3.1.6. Epidemiology of Post-traumatic Endophthalmitis

Post-traumatic endophthalmitis is a rare but devastating complication that includes risk factors such as the presence of an intraocular foreign body (IOFB), rupture of the lens, delayed repair of the primary globe, trauma with contaminated objects. The visual prognosis in post-traumatic endophthalmitis depends on the virulence of the microbe, the presence of detachment of the retina, the time of treatment, the presence or absence of an IOFB and the extent of the initial injury [38]. Post-traumatic endophthalmitis represents 25% to 30% of all endophthalmitis cases and its incidence was reported 10 times higher than post-surgical endophthalmitis [36,37,38,39]. Due to the lack of recent reports, we cannot estimate the prevalence of *Aspergillus* spp. etiology secondary to trauma. However, several authors reported that the incidence of fungal agents in post-traumatic endophthalmitis range from 0% to 15.4% [9,38,39]. Eye injuries with complications that degenerate into endophthalmitis are frequent in the workplace or in more rural areas where the main safety devices to protect the eyes are not properly used or not used at all [6,36,37,38,39]. In general, the risk of endophthalmitis is much greater with injuries produced by non-metallic foreign bodies, generally with contamination of microorganisms found in the soil, and especially when accompanied by crystalline lens lesions [38]. In addition, an increased risk of endophthalmitis has been reported following injuries from dental procedures, scratches from domestic and/or wild animals and from some food products [38,40]. In practice, mainly bacteria but also fungi are the major microorganisms responsible for the occurrence of post-traumatic endophthalmitis [2,3,4]. Therapeutic treatment and fundamentally prognosis are greatly influenced by the type of pathogenic microorganism involved, the nature of the lesion, the presence of IOFB and the geographic region in which ocular trauma occurs [3,34]. It should considered that the presence of a positive intraocular culture does not always lead to the development of endophthalmitis, in fact, in at least one third of the eyes subject to trauma and in the absence of endophthalmitis, bacterial growth has been demonstrated in intraocular fluids [38,39,40]. Therefore, it is crucial that all cases of samples positive to culture techniques must be suitably supported by clinical results [3,34]. While considering that the frequency of post-traumatic fungal endophthalmitis is much lower than that of bacterial origin, in the case of endophthalmitis due to injuries caused by accidents with trees and other vegetation, particular attention must be paid to exclude the involvement of fungal agents [3,34].

## 4. Clinical Features

EXFE clinical presentation is very variable, ranging from the classic endophthalmitis triad of decreased vision, red eye and ocular pain, to an insidious presentation with aspecific ocular findings and progressive vision loss [3,41,42]. Unlike bacterial endophthalmitis which usually has a hyperacute presentation, EXFE often presents with a latency period of weeks-months [3,34]. The intraocular inflammation in fungal endophthalmitis shows up in “clumps” within the aqueous and/or vitreous area, whereas intraocular inflammation is typically diffuse in bacterial endophthalmitis [2,3,41,42]. An intraocular infection has devastating consequences, leading to reduced vision and possibly irreversible blindness. Similarly, symptoms can be very different: vision loss can be mild for cases with peripheral vitreous lesions (snowballs and snowbanks) or severe for cases with great vitreous and/or anterior chamber inflammation [2,3,34]. Perikeratic reaction is a possible ocular finding as well as keratic precipitates, hypopyon and fibrinous anterior chamber (AC) exudation [3]. Scleritis has been reported as a presentation finding of EXFE following PPV [21,22,23].

## 5. Diagnosis

Diagnosis of exogenous endophthalmitis is based on clinical findings and supported by culture of vitreous or aqueous samples [3]. Negative cultures do not exclude the diagnosis of endophthalmitis since 20 to 30% of cases are culture-negative. Besides, molecular diagnostic techniques allow to provide an etiological diagnosis in many culture-negative cases [3,41,42,43]. The subacute onset of symptoms, such as red eye and vision loss, following eye infection, eye surgery or trauma is suspicious for EXFE; however, a hyperacute presentation has been also reported, mainly following keratoplasty [3,34]. Once established the suspect of exogenous endophthalmitis, it is necessary to obtain both an AC tap and a vitreous tap before initiating any antimicrobial therapy. In cases of suspected EXFE correlated with keratoplasty of keratomycosis, a corneal scraping is recommended [3,41,42]. The collection of vitreous humor is considered the most reliable and most sensitive method for FE diagnosis [3,41,42]. Vitreous humor samples can be obtained either via posterior chamber needle aspiration or via therapeutic vitrectomy, being the latter method the most reliable for fungi identification [41,42]. Therapeutic vitrectomy should be performed in aseptic conditions by a specialized ophthalmologist. Samples should be always evaluated for both fungi and bacteria, using a wide-ranging polymerase chain reaction (PCR) system, it is possible to detect the fungal and bacterial genome in the ocular fluids (aqueous humor or vitreous fluids) of patients with fungal endophthalmitis [41,42,43]. Being PPV a traumatic procedure, vitreous needle aspiration is more commonly used for obtaining vitreous samples, even if some reviews show substantial benefits in PPV for the management of FE [21,22,23].

## 6. Pharmacokinetics and Pharmacodynamics of Antifungals in EFE

Characteristics of intravitreal and systemic antifungal drugs commonly used in EXFE are shown in Table 1.

### 6.1. Intravitreal Antifungals

Once an anti-infective solution is injected into the vitreous humour, the initial concentration of the anti-infective in the vitreous cavity depends on the extent of its distribution and the initial dose: shortly after intravitreal administration, high levels of antifungal drug reach inside the eye and the distribution is notably affected by characteristics of molecule used, injected dose, eye physiology (e.g., rate of elimination) [44,45].

#### 6.1.1. Intravitreal Polyenes in Exogenous Fungal Endophthalmitis (EXFE)

Amphotericin B deoxycholate (DAmB) is the cornerstone of antifungal intravitreal therapy in EXFE, with powerful activity against *Aspergillus* colonies and low risk of intrinsic or developing resistance during treatment [46]. DAmB covers a wide variety of fungi, including moulds such as *Aspergillus* spp. that are generally susceptible, *A. terreus* and the clinically rare *A. alliaceus* [44,45,46]. Minimum inhibitory concentrations (MICs) vary according to the species: A. fumigatus and A. niger generally have lower MICs than A. flavus and A. versicolor [44,45,46,47]. DAmB is marked by a dose-related ocular toxicity, reported commonly for intravitreal doses greater than 25 µg, with risk of severe intraocular inflammation, retinal necrosis, and cataract formation. Intravitreal shot of 5 to 10 µg of amphotericin B deoxycholate in 0.1 mL solution has been considered safe in vitrectomised and non-vitrectomised eyes [46,47]. There is no standardization about the number or frequency of repeated injections. This depends on the clinical response of the eye after initial injection, taking also into account possible side effects due to intraocular inflammation. Its high molecular weight (over than 500 Da) reduces the migration through vitreous humor meshwork and the diffusion in the posterior chamber [43,44,47]. DAmB showed a vitreous half-life of 6.9 to 15.1 days (in rabbit studies), with a faster washout in vitrectomized eyes [46,48]. Physiological conditions (e.g., age) and pathological alterations affecting the vitreous could change its distribution. Net charge of anti-infective agents at the vitreous pH could lead to a decrease in drug diffusivity because of negatively charged meshwork of the vitreous; nevertheless, DAmB was reported to have a mild negative net charge (−0.02) [44,45,47]. Another factor is age-related or iatrogenic liquefaction of humor vitreous: theoretically, liquefaction is responsible for an increase in drug diffusivity and convection flow, but there is no evidence that liquefaction changes the pharmacokinetic of intravitreal drugs [46,47]. On the other side, vitrectomy could reduce the half-life of DAmB with a four-fold increase in drug elimination; the half-time decreases to 1.4–1.8 days half-time in aphakic plus vitrectomized eyes [44,45,46,48].

#### 6.1.2. Intravitreal Azoles in EFE

Most of the azoles possess a high degree of lipophilicity, with poor solubility in an aqueous medium: this feature is linked to their structure with aromatic rings conveying a lipophilic character [42,43]. Voriconazole can boast a wide efficacy against yeasts and molds, in fact, *Aspergillus* spp., *Blastomyces dermatitidis*, *Candida* spp., *Coccidioides immitis*, *Cryptococcus neoformans* and *Histoplasma capsulatum* are all microorganisms that can be effectively inhibited by voriconazole. In addition, intravitreal voriconazole injections have been clinically effective in treatment of fungal endophthalmitis caused by *Scedosporium apiospermum* and *Fusarium* species [49]. In a recent retrospective study on fungal endophthalmitis, all 47 fungal isolates from eyes resulted sensitivity to intravitreal voriconazole, whereas only 69% of them were sensitive to intravitreal DAmB [50,51]. Levels of voriconazole in the first 8 h exceed about 10 times the MIC for fungal organisms causing endophthalmitis, as demonstrated in several PK studies [44,45]. Nevertheless, since the half-life of voriconazole in the vitreous is only 2.5 h, the real concentration of the drug declines precipitously making it essential to repeat injections in order to maintain an adequate therapeutic level [42,43,44,45,50,51]. At the intravitreal level in in vivo models, a lower risk of retinal toxicity was associated with the use of voriconazole compared to the intravitreal DAmB. In fact, up to high concentrations (such as 25 µg/mL) there were no histopathological or electroretinographic changes in the rat retinas [50,51]. Only at concentrations of 50 µg/mL or higher specific areas of necrosis could be highlighted [50,51], so it can be safely assumed that this drug can be used in humans up to even higher concentrations (up to at 100 µg) [44,45,50,51].

Despite data on real-life use in this field are scarce, a promising alternative to voriconazole may be isavuconazole, a novel broad-spectrum azole [52]. Guest et al. reported that isavuconazole was effective in treating experimental fungal endophthalmitis in mice due to *A. fumigatus*: isavuconazole treatment via all routes (oral and/or intravitreal) reduced fungal burden in *A. fumigatus* infected eyes [52].

#### 6.1.3. Place in Therapy of Intravitreal Echinocandins

Kusbeci et al. [53] studied in rabbits the efficacy of intravitreal injection of caspofungin (0.1%) considering parameters of fungal load and histopathology, before and after the treatment. A statistically significant difference between scores was found between the saline (control) and the caspofungin-treated group [53]. This demonstrated the effectiveness and safety of caspofungin (0.1%) in fungal endophthalmitis. However, like voriconazole, concentrations rapidly decreased due to exponential decay, despite having a longer half-life of 6.28 h [54]. Caspofungin proved effective in treating post-cataract surgery endophthalmitis [54]. Besides, caspofungin along with amphotericin B was effectively used in treating postoperative endophthalmitis caused by *A. flavus* in a diabetic patient [55].

### 6.2. Systemic Antifungal Therapy

The blood–ocular barrier with its tight endothelial junctions inhibits the movement of high molecular weight antifungal drugs such as AmB, especially when administered parenterally [44,45]. Low aqueous solubility halts its penetration in ocular anatomical barriers such as cornea and blood-retinal barrier [44,45]. To improve the ocular penetration and safety of AmB, lipoidal formulations containing AmB have been studied [47,56]. In particular, a liposomal amphotericin B (L-AmB) formulation and an amphotericin B lipid complex (ABLC) have been investigated. Both formulations were observed to penetrate the blood–retinal barrier in inflamed eyes, but the concentration of AmB in the aqueous humor following L-AMB administration was 8-times more than the concentration of the amphotericin B lipid complex (ABLC) and DAmB [42,43,44,56]. Voriconazole showed good ocular bioavailability when used systemically (oral or intravenous), achieving therapeutic levels in both the vitreous and the aqueous humor [56,57]. In exogenous mold endophthalmitis, vitrectomy and intraocular injections of antifungal agents (amphotericin B deoxycholate or voriconazole) alone may suffice, but the addition of systemic voriconazole is almost always indicated unless the fungus is known to be resistant to azoles [56].

### 6.3. Surgical Treatment

EXFE is a rare intraocular infection with potentially devastating ocular complications. A prospective trial on EXFE is impossible due to its rarity, the presence of predisposing factors and the different causal organisms. Different studies have used different surgical techniques, including different timing, tool caliper, concurrent use of systemic or intravitreal therapy, and different tamponade, making it impossible to draw definite conclusions. Vitrectomy is certainly useful as a diagnostic tool because it yields significantly higher rates of positive cultures [55]. Furthermore, it is known that early vitrectomy with concurrent use of intravitreal injections seems to hold the best outcomes and that use of antifungal and antimicrobial therapy may be warranted in suspected cases [57,58]. Although some authors suggest that silicone oil should be used in any case, others do not agree because silicone may play a weaker role as fungistatic compared to its bacteriostatic properties [57]. Additionally, it may change the concentration of subsequent intravitreal injections of antifungal agents with a potential risk of retinal toxicity [37]. Gao et al. suggested a role for therapeutic penetrating keratoplasty in keratomycosis-related EXFE, for both removing the infection focus and allowing a better evaluation of posterior structures [36]. The mean time between onset of antifungal therapy and penetrating keratoplasty was 4.5 days and the concomitant intravitreal injection of antifungal agent was performed in all cases [36].

## 7. Conclusions

**Figure 2:**
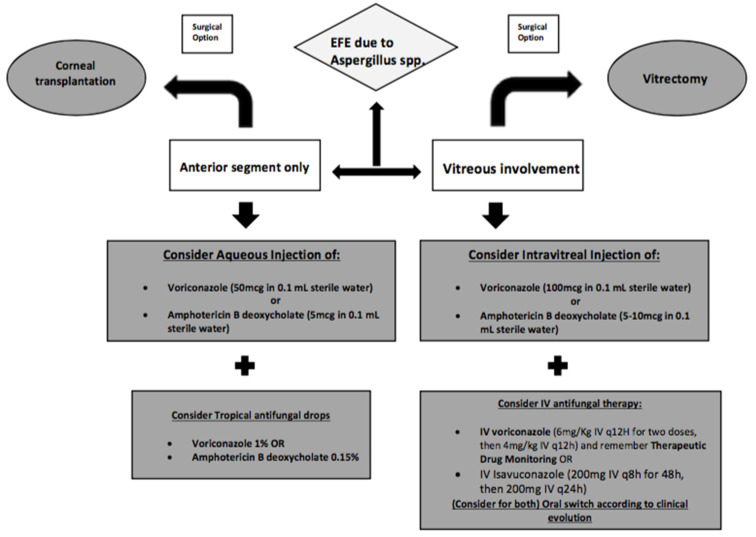
Practical guidelines for clinical management of exogenous fungal endophthalmitis.

EXFE due to *Aspergillus* spp. may theoretically present with proteiform clinical features, with delayed diagnosis and risk of poor visual outcomes. Moreover, medical treatment has several limitations both in empirical and target therapy. Empirical coverage collides with scarce data and low-quality evidence on antifungal penetration and dosage in local and systemic therapy for EXFE. In Figure 2 we provide shared practical guidelines to simplify clinical decision making (Figure 2). On the other hand, target therapy is not always possible for high rates of negative-culture endophthalmitis and lack of fungal susceptibility testing. In our opinion, strict cooperation between clinical microbiologists, infectious diseases specialists and ophthalmologists in this process may allow the best practices to be optimized, highlighting main steps for a rapid diagnosis and management. This review may pave the way for larger studies to improve the clinical management of exogenous fungal endophthalmitis.

## Figures and Tables

**Table 1:** Characteristics of main systemic and intravitreal antifungal drugs used for exogenous fungal endophthalmitis.

Route of Administration	Class of Antifungal	Drug	Spectrum	Rate of Antifungal Resistance	Diffusion	Half-Life	Toxicity
Intravitreal	Polyenes	Amphotericin B	Very wide for Moulds and *Candida* spp.	Very Low	Low-moderate (High molecular weight, negative charge)	1.4–15.1 days	Ocular, Dose related (<25 ug)
	Azoles	Voriconazole	Wide for *Aspergillus* and *Candida* spp.	Low for *Aspergillus*, Low-moderate for *Candida* spp.	High	2.5 h	Ocular, Dose related
		Isavuconazole	Wide for *Aspergillus*, *Mucor* and *Candida* spp.	Low (limited data)	High (Limited data)	NA	NA
	Echinocandins	Caspofungin	Wide for *Candida* spp., Less for *Aspergillus* spp.	Low for *Candida* spp.	Low-moderate	6.2 h	NA
Systemic	Polyenes	Amphotericin B	Very wide for Moulds and *Candida* spp.	Very Low	Low	153 h	Kidney, infusion-related, Na, K, Mg
	Azoles	Voriconazole	Wide for *Aspergillus* and *Candida* spp.	Low for *Aspergillus*, Low-moderate for *Candida* spp.	High	6 h	Visual, Kidney, GI, Skin, Na, K
		Isavuconazole	Wide for *Aspergillus*, *Mucor* and *Candida* spp.	Low (limited data)	High	4–7 h	GI, Kidney, Na, K

Footnote: NA: not applicable.
